# Situational and Age-Dependent Decision Making during Life Threatening Distress in *Myotis macrodactylus*

**DOI:** 10.1371/journal.pone.0132817

**Published:** 2015-07-16

**Authors:** Xiaobin Huang, Jagmeet S. Kanwal, Tinglei Jiang, Zhenyu Long, Bo Luo, Xinke Yue, Yongbo Gu, Jiang Feng

**Affiliations:** 1 Jilin Provincial Key Laboratory of Animal Resource Conservation and Utilization, Northeast Normal University, Changchun, China; 2 Key Laboratory for Wetland Ecology and Vegetation Restoration of National Environmental Protection, Northeast Normal University, Changchun, China; 3 Departments of Neurology, Neuroscience and Psychology, Georgetown University Medical Center, Washington DC, United States of America; Arizona State University, UNITED STATES

## Abstract

Echolocation and audiovocal communication have been studied extensively in bats. The manner in which these abilities are incorporated within escape behaviors during life-threatening distress is largely unknown. Here we tested the hypothesis that behavioral response profiles expressed during distress are relatively stereotypic given their evolutionary adaptations to avoid predators. We subjected juvenile and adult big-footed myotis (*Myotis macrodactylus*) to a sequence of three types of life threatening distress: 1) trapping them in a mist-net (environmental threat), 2) approaching them when trapped (predator threat), and 3) partially restraining their freedom to move (arrest), and recorded their escape behavior in each of the three conditions. Response profiles differed across individuals and with the context in which they were expressed. During environmental and predator threat, bats displayed significantly more biting and wing-flapping behaviors and emitted more echolocation pulses than during arrest. Response profiles also varied with age. During arrest, juveniles were more likely than adults to emit distress calls and vice-versa for biting and wing flapping during environmental and predator threat. Overall, individualized response profiles were classified into ten clusters that were aligned along two divergent response trajectories when viewed within two-dimensional, multifactorial decision space. Juvenile behaviors tended to follow a predominantly “social-dependence” trajectory, whereas adult behaviors were mostly aligned along a “self-reliance” trajectory. We conclude that bats modify their vocal behavior and make age-appropriate and contextually adaptive decisions when distressed. This decision-making ability is consistent with observations in other social species, including humans.

## Introduction

Given its significance within evolutionary, ecological and neuroethological contexts, bats’ predatory behavior [1,2,3,4] and related neural adaptations for echolocation [5,6,7,8,9] are well studied. So are the escape behaviors, or “antipredator tactics” of their prey, largely insects [10,11]. Studies of bats’ own escape behavior and how they employ their echolocation and audiovocal communication abilities under life threatening situations, however, are rare [12,13]. To actively avoid predation, bats may voluntarily adjust certain routine behaviors, such as the choice of day roosts [14] and the timing of departure from a roost [15]. This type of decision making to avoid distress is facilitated by auditory [16], visual [17], and chemical cues [18]. We know even less about on the spot, “impulsive” decision making when bats need to escape from a life-threatening situation. It is possible that such decision-making in bats is purely reflexive, given their flight and foraging adaptations that naturally insulate them from their predators.

Most animals face the risk of being preyed upon by another species. In most species, antipredator tactics are displayed within two contexts, either to avoid being captured or to increase the probability of escape if already caught [19]. Previous studies on antipredator tactics have focused mainly on the first context. These studies show that prey adjust their escape tactics based on the context in which they encounter the predator, including the level of urgency and environmental factors (e.g., temperature and food availability) [20]. The level of urgency usually is determined by the immediate presence or absence of the predator, predator behavior, and distance to prey. In herbivores, immediate presence of predators and the distance to predator affect the time allocation to vigilance vs. drinking among individuals [21]. Also, many animals adjust the acoustic structure and parameters of distress or alarm calls based on predator types, location and behavior [22,23,24].

Antipredator tactics also vary among individuals based on their sex [25], experience [26], group size [27] and age [28], and possibly other physiological and experiential factors. Juveniles usually are less experienced with predator avoidance than adults [28]. Despite abundant evidence for the emission of distress calls [29,30], a systematic investigation of behavioral responses during environment or predator induced distress in bats is lacking. Here, we tested the hypothesis that behavioral responses, initiated by the sudden onset of distress or fear, are relatively stereotypic and reflexive in microchiropteran (mostly insectivorous) bat species given their evolutionary adaptations to avoid predators. Alternatively, given the common decision-making circuitry present in all mammalian brains [31, 32], bats may exhibit context-dependent expression of fear and aggression, typical of a flight or fight response encountered in most other species [33]. They may “choose” between several possible behavioral responses based on their potential benefit and cost within a given situation or context [34]. We also hypothesized that in bats, age may not make a significant difference in their choice, given their relative lack of experience with predators at the individual level and the evolutionally established strategies of flight and a relatively protective cave habitat for predator avoidance.

We selected big-footed myotis (*M*. *macrodactylus*) to test our hypotheses given their crepuscular and nocturnal, insectivorous feeding habit and the opportunity to capture them for ongoing ecological studies. To test an individual’s reaction to distress, we employed restraint by trapping bats in a mist-net (environmental threat), direct approach by a human (predator risk or threat) and finally, predator restraint by grabbing an individual (arrest). We obtained audio and video recordings of escape behaviors during each of these three life-threatening conditions. We discovered that big-footed myotis routinely adjust their behavioral responses according to the type/level of distress associated with each situation. Also, the probability of eliciting each of these behaviors in each situation varied with an individual’s age. We discuss these unexpected results from a comparative behavioral and neurobiological perspective.

## Materials and Methods

### Study site and animal

Our study was conducted at Dalazi Cave (125°50′9.8″E, 41°3′55.8″N) in Ji’an, Jilin Province, China. This cave has a big size (ca. 80 × 12m and 7 m high) and wide entrance (ca. 8 × 2 m), and houses about 500 *M*. *macrodactylus*. We conduct ecological research every year at this location.

*M*. *macrodactylus* is a medium-sized myotis (Vespertilionidae) [35], and emits frequency-modulated (FM) echolocation signals. Previous studies showed that female *M*. *macrodactylus* give birth in early July, and young bats can fly at around 32 days [36]. Thus, we carried out our experiment from late July to early September 2013. In this study, we only captured juvenile bats and female adults by mist nets because of the dispersion of almost all of male adults from the maternal colony. Individuals with unfused phalangeal epiphyses were classified as juveniles, those with fused phalangeal epiphyses as adults (unknown age) [37].

### Experimental procedure

**Fig 1:**
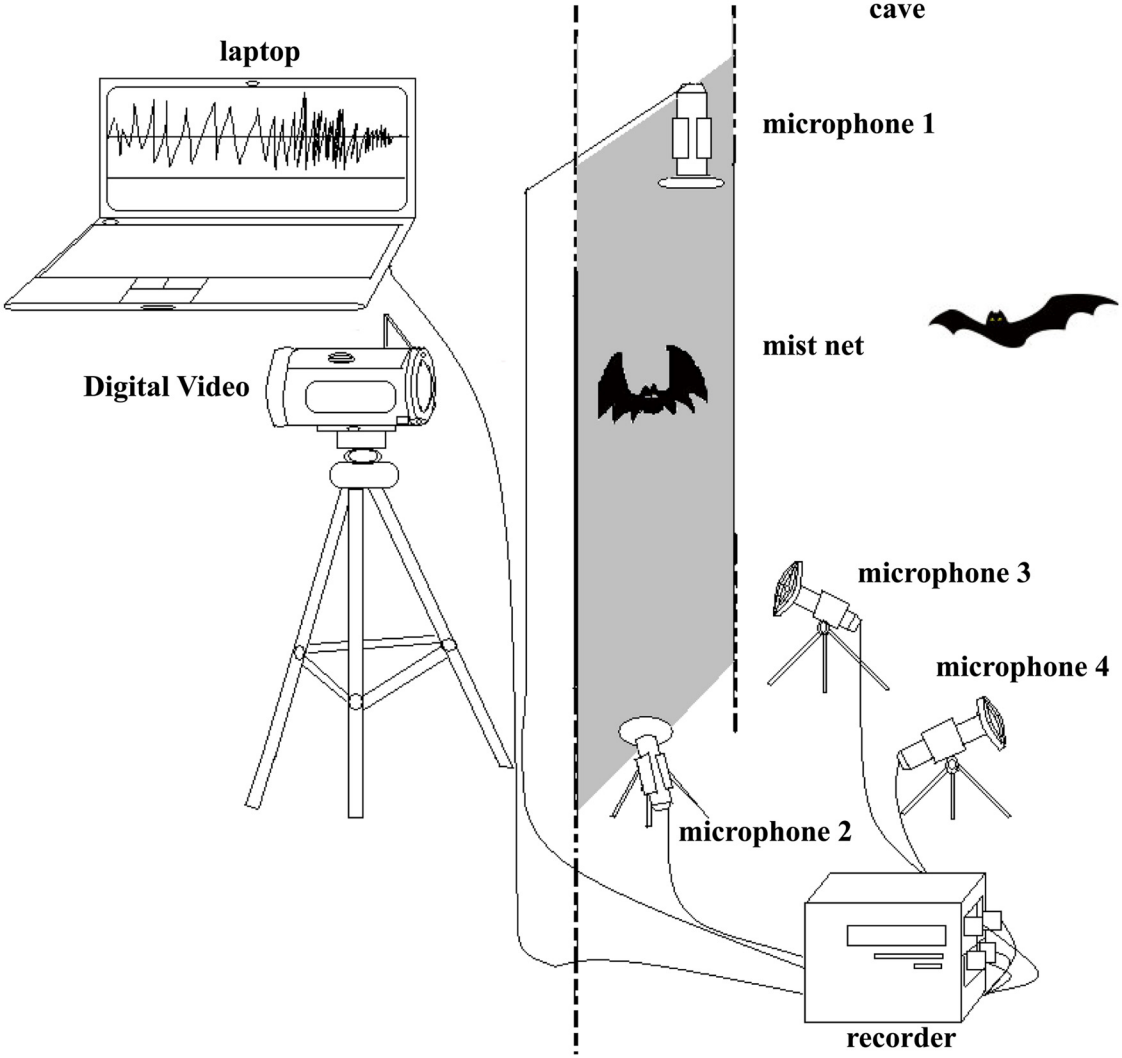
Schematic of experimental setup. Bats were trapped in mist-nets. An infrared digital video camera monitored the behavior of trapped bats. Three condenser microphones (Avisoft Bioacoustics CM16/CMPA) synchronously recorded the calls of trapped bats in different directions, microphone 4 recorded the calls of other bats in cave. All microphones connected with an ultrasonic acquisition system, and all calls were recorded onto a Dell Latitude laptop.

We set out our experimental apparatus (Fig 1) at the cave entrance about 1 h before sunset. In this experiment, bats were captured in mist nets (ca. 2 × 2 m). A part of the cave entrance was sealed with a gobo (ca. 4 × 2 m) in order to increase the capture rate. We initiated the experimental procedure when we detected a bat trapped by mist net. A researcher (about 2 m away from the trap) operated an infrared digital video camera (Sony Handycam HDR-CX760E, Japan) to record the behavior of the trapped bat. We used an ultrasonic acquisition system (Avisoft-UltraSoundGate 416H, Avisoft Bioacoustics, Berlin, Germany) to synchronously record the calls of the trapped bat, with a sampling frequency of 375 kHz and 16-bit resolution. This ultrasonic acquisition system, connected with a laptop (about 5 m away from the trap), continuously recorded vocal activity for the duration of the trial. A second researcher (about 10 m away from the trap) directly approached the trapped bat with heavy footsteps and flashlight to convey detection and intent to capture [38], then captured and held gently the bat in the left hand (by right handed researcher). The researcher gently pressed between the shoulder blades on the back with the thumb and softly held on to the bat’s chest with other fingers, making sure that its head, feet and wings were free to move. Each bat was handled in precisely the same way. The first researcher kept quiet as much as possible to reduce interference during the experiment. If two or more bats were simultaneously trapped in the mist net, the trial was discarded.

For each individual, we sampled behavioral responses during three conditions that represented different types/levels of threat, namely, environmental threat (restraint by trapping bats in a mist-net), predator threat (perceived deliberate and gradual approach by a human) and arrest (physical restraint from holding on to the individual). Each condition lasted for about 30s except for approach, which was normally less than the other periods because it was difficult to control given that some bats escaped before being captured. This paradigm has been effective for studying antipredator behavior in several other species [26,39].

Captured bats were identified to species, sexed, and aged [37,40], then marked using a numbered alloy band with 2.9 mm diameter (Porzana Ltd, UK) on the right forearm. Every bat was put in a cloth bag, and released at the end of each (daily) trial. Each bat was tested only once; if a bat was captured again, the trial was rejected. Trials usually lasted for two hours (ca. 19:00–21:00) in good weather conditions. To minimize interference, we conducted our experiment every three days, for a total of 14 nights. Altogether, we obtained recordings from 109 different bats (mean ± SD every night 7.79 ± 2.39).

### Behavioral and acoustic analysis

We excluded some individuals from the analysis for which either we did not get clear videos or we were unable to distinguish their calls from those of other bats. Also, if a bat failed to demonstrate free and unimpeded wing movements, it was excluded from the study. All other animals were included in the study. Altogether, we obtained behavioral and acoustic data from 82 *M*. *macrodactylus* (54 adult females and 28 juveniles) under each of the three conditions (Table 1). We defined four common behavioral responses by a careful (frame-by-frame) analysis of the audio and video recordings.

1. Biting: Rapid mouth opening and closing jaw movements resulting in clasping of the mist-net or attempting to do the same with the researcher’s hand. However, bats probably perceive differently in the two situations.
2. Wing flapping: up-and-down motion of bat’s partially spread out wings, probably representing an attempt to escape. Each up-and-down flapping cycle was counted as one occurrence.
3. Vocalizations: Sound files from the microphone closest to the target animal were used to minimize interference from other bats’ vocalizations. The sound analysis was used to detect the occurrence of echolocation pulses and distress syllables within each context of life threatening distress. Only echolocation and distress vocalizations with a high signal-to-noise ratio were counted. Spectrographic identification was performed in Avisoft-SAS Lab Pro, 4.3 software (Avisoft Bioacousics, German; 512 FFT, 93.75% overlap, Hamming window). We measured the duration, interelement interval, peak frequency and bandwidth of echolocation pulses and distress syllables. The definition and classification of syllables followed the terminology adopted by Kanwal et al. [41] and Ma et al. [42].
4. Freezing: Bats remained motionless with their wings either folded or spread out. No other behaviors and vocalizations were expressed at this time.

**Table 1:** Escape behavior of big-footed myotis both in adults (N = 54) and juveniles (N = 28) during three contexts.

Behavioral variables	Environmental threat		Predator threat		Arrest	
	Adult	Juvenile	Adult	Juvenile	Adult	Juvenile
Proportion of time biting (%) ^a^	91.89 ± 1.95	68.64 ± 7.91	56.76 ± 4.55	44.11 ± 7.14	1.06 ± 0.54	1.43 ± 0.99
Number of flapping/second ^a^	0.31 ± 0.04	0.22 ± 0.07	0.52 ± 0.07	0.36 ± 0.08	0.06 ± 0.01	0.06 ± 0.02
Number of pulses/second ^a^	1.95 ± 0.33	2.02 ± 0.59	1.12 ± 0.28	0.94 ± 0.29	0.30 ± 0.10	0.22 ± 0.10
Number of calls/second ^a^	0.02 ± 0.016	0.01 ± 0.007	0	0	1.05 ± 0.23	2.06 ± 0.47
Syllable frequency/call ^a^	0.24 ± 0.17	1.08 ± 0.63	0	0	3.04 ± 0.48	4.41 ± 0.69
% exhibiting freezing behavior ^b^	0.00	10.71	3.70	25.00	25.93	17.86

^a^ Data are presented as Mean ± SE.

^b^ Data are presented as percentage.

‘0’ indicates that big-footed myotis didn’t emit distress calls during predator threat.

In addition to the above inclusion and exclusion criteria, we obtained comparable measures of each response included in the study. We calculated the biting propensity (Biting; proportion of time within each context during which the animal was engaged in biting), wing flapping rate (WFR; number of flapping cycles/s), the echolocation pulse rate (EPR; number of echolocation pulses/s), the distress call rate (DCR; number of distress calls emitted/s) and the total number of distress syllables within each call (DS/call). We also calculated the percentage of bats that remained motionless, which was used as a measure of freezing response (FR; % exhibiting freezing behavior).

### Statistical analyses

We used multivariate, nonparametric approaches to clustering (*k*-means clustering) and linear discriminant analysis (LDA; JMP software, SAS Inc.) for identifying and defining escape tactics adopted by juvenile versus adult bats under different conditions of threat. LDA extracts a linear combination of features that characterizes or separates two or more classes of objects or events. LDA explicitly attempts to model the difference between the classes of data. The resulting combination is typically used either as a linear classifier or for dimensionality reduction before later classification.

We used hierarchical and *k*-means clustering (using a principal components analysis algorithm) to identify the minimum number of clusters required to obtain a good classification of all the data based upon the six response variables (Biting, WFR, EPR, DCR, DS/call, FR) observed in both juvenile and adult bats. *k*-means clustering partitions the data space such that each observation belongs to the cluster with the nearest multivariate mean or cluster center. Results were visualized as a scatter plot for the first two principle components. Parallel coordinate plots, a common graphical facility for visualizing geometrical patterns in multivariate data, were used to identify the response patterns underlying each cluster. A parallel coordinate plot typically consists of a polyline in n-dimensional space connecting points on “n” parallel, vertical and equally spaced axes; the position of the vertex on the i-th axis corresponds to the i-th coordinate of the point.

LDA was performed to test the effect of sex, age and response types under three contexts both in adult and juvenile bats. Mahalanobis distance was used to determine the fit of a data point from each group’s multivariate mean depicted by the center of the ellipses corresponding to a 95% confidence limit (CL) for the mean. Significance is indicated by a lack of intersection between adjacent ellipses for each group. Either a linear or a quadratic fit was used to minimize misclassification of the data. All measured parameters were included in the final model after testing with stepwise model fitting.

Since the data on proportion of time biting, WFR, EPR, DCR and the total number of syllables within each call (DS/call) were not normally distributed (Kolmogorov-Smirnov test, *P*< 0.05), we also performed multiple comparisons using Friedman tests followed by Dunn's multiple comparisons tests. For the percentage of bats remaining motionless, Pearson’s chi-square test was performed, and Fisher’s exact test was used for *post hoc* pairwise comparisons. Moreover, in order to assess the differences in escape behaviors between adult and juvenile bats during each context, Independent-Sample T test or Mann-Whitney U test was performed for proportion of time biting, or for WFR, for EPR and for DCR depending on the distribution of data. Fisher's exact test was performed for the percentage of bats remaining motionless. We compared the difference of acoustic parameters between echolocation pulses and distress syllables by Mann-Whitney U test. All classic statistical tests were performed using either SPSS 17.0 or GraphPad Instat version 3.0. All tests were two tailed at a significance level of 0.05.

### Ethics Statement

Our work did not cause any physical injuries to bats. All research was conducted according to the relevant laws for experiments involving vertebrates of the People’s Republic of China, and approved by the Forestry Bureau of Jilin Province of China and the National Animal Research Authority in Northeast Normal University, China (approval number: NENU-W-2008-108).

## Results

### Acoustic analysis

**Fig 2:**
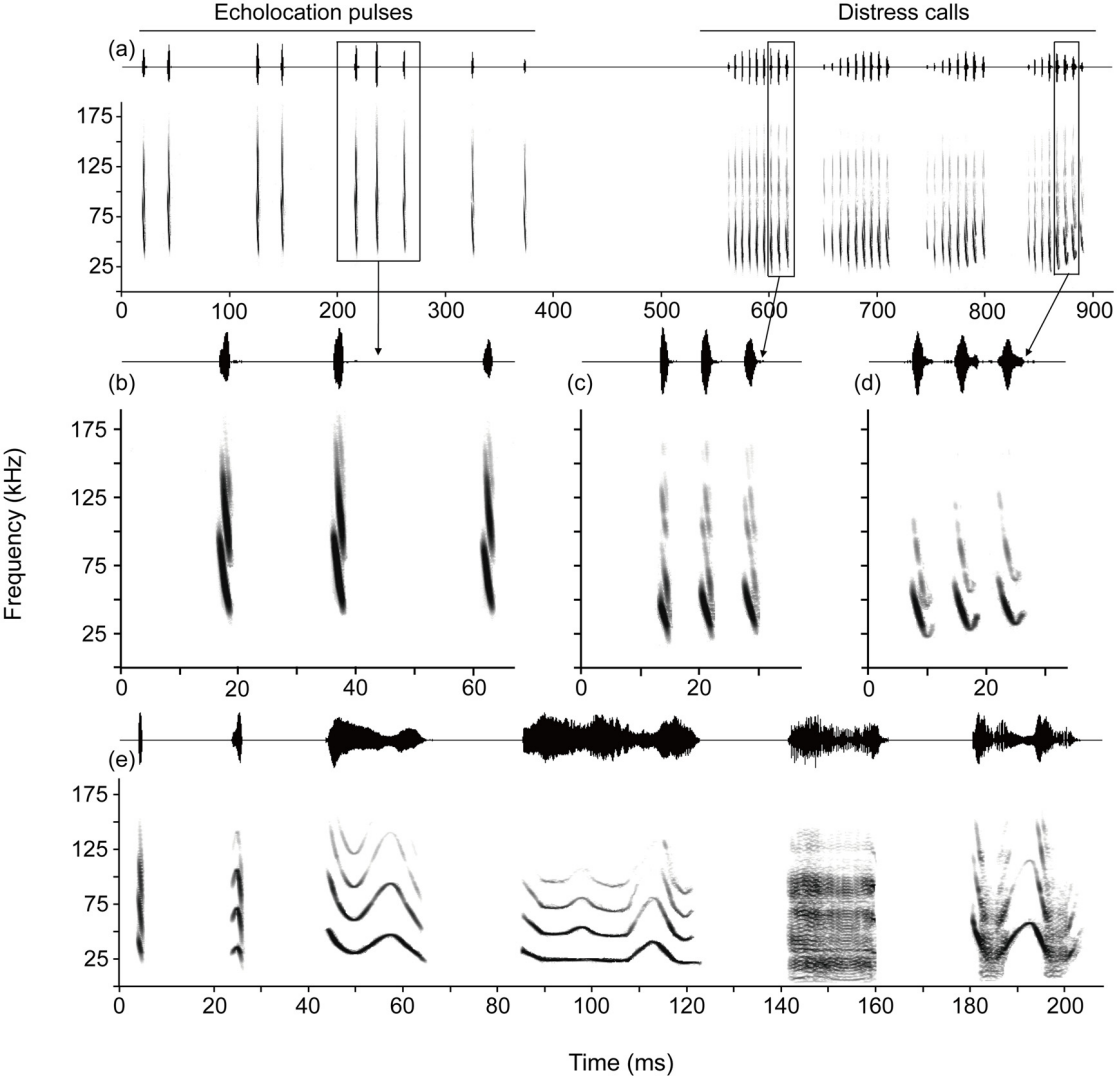
Sound envelopes (upper trace) and spectrograms (lower trace) of echolocation pulses and distress calls emitted during distress in *M*. *macrodactylus*. (a) Echolocation pulses and syllable trains emitted as a sequence within a vocalization. (b) Echolocation pulses shown on an expanded time scale to show spectrographic pattern of each pulse. (c) and (d) Two of the most common distress syllables shown on an expanded time scale to show spectrographic pattern of each syllable. (e) Representative samples of six additional types of single syllables present within distress calls, demonstrating the spectrographic richness.

Vocalizations emitted for echolocation and during distress generally consist of a stream or “train” of individual sounds that have been identified as “pulses” and “syllables”, respectively [6,41]. There was no significant difference in bandwidth between syllables present within distress calls and echolocation pulses (Mann-Whitney *U* = 2898.00, *P* = 0.51). The syllables present within distress calls, however, were significantly different from echolocation pulses along several other acoustic parameters (Fig 2), so that distinguishing between the two was not a problem. In particular, distress syllables had a longer duration (Mann-Whitney *U* = 2371.50, *P* = 0.012), shorter inter-syllable interval (Mann-Whitney *U* = 81.00, *P* < 0.001), and lower peak frequency (Mann-Whitney *U* = 191.00, *P* < 0.001) compared to individual echolocation pulses. Moreover, syllables present within distress calls exhibited a large variation in their acoustic structure compared to the relatively stereotypic echolocation pulses. Amplitude envelopes and spectrograms of different representative examples for each type of vocalization are shown in Fig 2.

Distress calls were the main vocalizations (adults: 48.15%; Juveniles: 64.29%) emitted during arrest whereas echolocation pulses were mostly emitted during environmental (adults: 90.74%; Juveniles: 78.57%) and predator threat (adults: 75.93%; Juveniles: 57.14%). Only a few individuals emitted distress calls when trapped in the net (adults: 3.7%; juveniles: 10.71%), and none of them emitted distress calls during predator approach.

### Behavioral response profiles

Each animal when tested for its behavioral responses under all three conditions, yielded a response (“personality”) profile consisting of a specific combination of escape response measures. Using binary logic, we estimated that the presence or absence of six categorically different responses, measured under three separate conditions, can generate ((2)^6^)^3^ or 262,144 different combinations of outcomes, assuming the same combination can be present in each of the three conditions. However, since the emission rate of distress calls is closely related to the number of syllables within each distress call (i.e. DS/call), these can be pooled into one response variable. Similarly, wing flapping and staying motionless can be considered as mutually exclusive responses. Assuming the presence or absence of only 4 possible responses of bats under all three contexts of life-threatening distress, it is still possible to generate up to ((2)^4^)^3^ or 4,096 different response profiles. Since five of the six response variables, however can vary along a continuum, the potential number of different response profiles is much larger.

**Fig 3:**
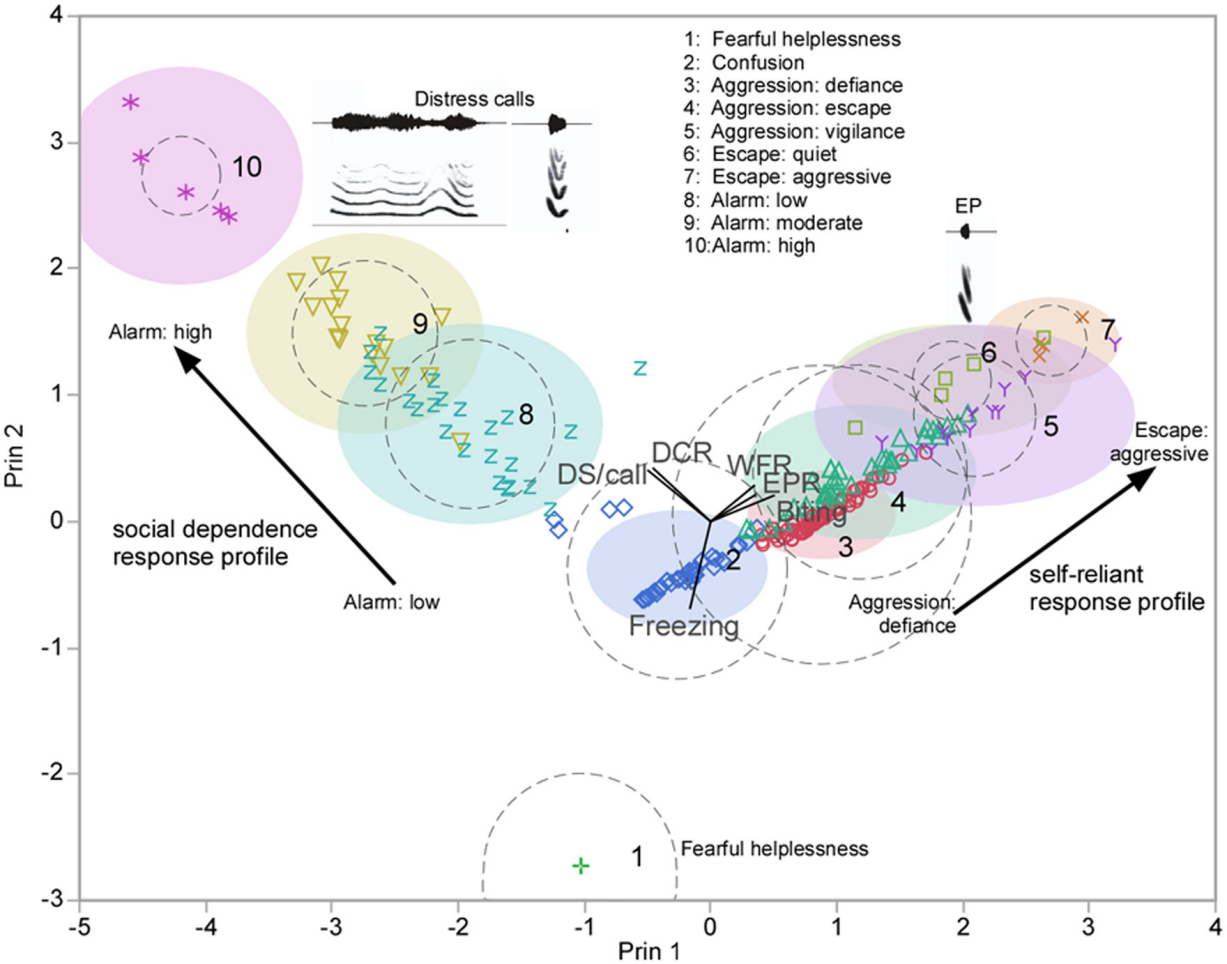
*K*-means cluster plot showing different behavioral approaches adopted (for coping with life-threatening distress experienced by an individual) within multivariate decision space. Plot is of points and clusters in the first two principal components. Circles are drawn around the cluster centers. The sizes of the circle outlines are proportional to the count inside the cluster. The shaded areas are the 90% density contour around the mean, indicating where 90% of the observations in that cluster would fall. Data values belonging to each cluster are indicated by different colored symbols. Behavioral classification was based upon examination of coordinate plots characterizing each cluster. Ray plot in the middle indicates contribution of each parameter. Biting, distress and freezing behaviors were roughly equally weighted and showed orthogonal mapping within the plot.

In stark contrast to theoretical considerations, optimal parsing of the data (maximal reduction per step in the unexplained variation) via automated clustering using a hierarchical branching tree algorithm revealed only 10 clusters. A best fit index based on the cubic clustering criterion obtained from iterative clustering using the robust normal mixtures method independently revealed as well that 10 clusters produce the best fit for the data when tested independently for adult and juvenile bats. Therefore, in our final analysis, we seeded a *k*-means clustering algorithm to generate clusters for 10 response profiles, independent of context and age of the individual. A scatterplot with data points color-coded for each cluster produced from this analysis revealed either separate or partially overlapping but distinct clusters organized as two divergent trajectories of cluster locations within either a two or three-dimensional space (Fig 3). Response profiles of two juvenile males were indicated by a decluttering procedure to be outliers and were eliminated from this analysis. Furthermore, similar clustering methods applied independently to the data segregated by condition also revealed a range of 8 to 10 clusters (not shown) as providing the best fit for each dataset.

**Fig 4:**
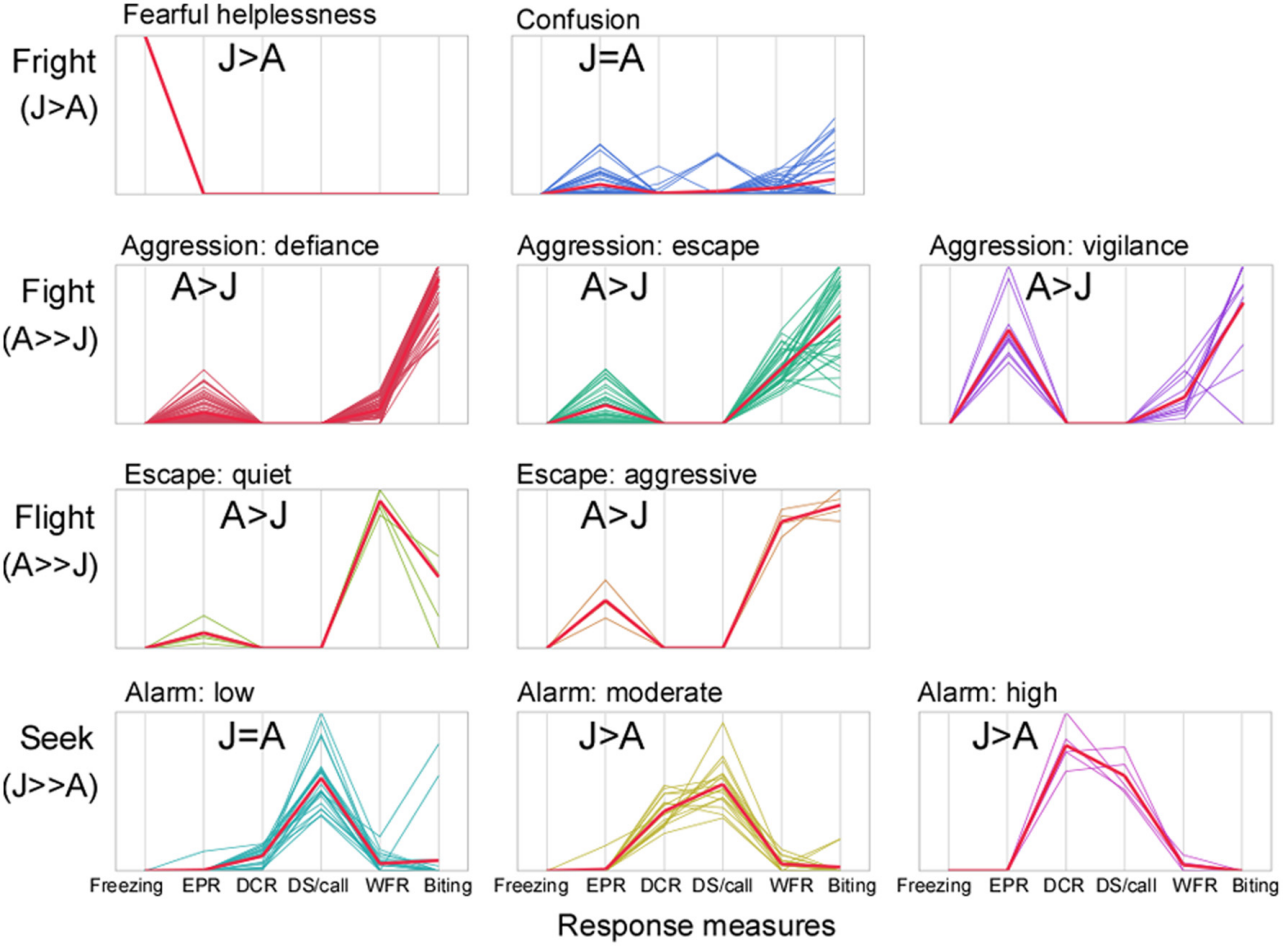
Context-independent classification scheme of escape response profiles expressed when coping with life-threatening distress. This scheme is based upon parallel coordinate plots obtained from multivariate cluster analysis used to categorize each combination of escape behaviors elicited. The prominence of each behavior varied with age (A refers to Adult, J refers to Juvenile) and distress situation. The heavy line represents the average and the vertical axis represents all variables scaled to the same length.

Parallel coordinate plots provided a useful visual representation of the composite profiles for each of the ten response combinations (Fig 4). These plots revealed a progression of behaviors from those eliciting assistance via emission of distress calls to those involving aggressive tactics to escape a distressful situation. We grouped the identified clusters of response profiles according to the common “fight” and “flight” response of animals. We also identified states expressing fright and seeking assistance, which we considered as being distinct from active flight or fight. As shown in Fig 4, each type had further sub-categories based on the exact combination of behaviors expressed by each individual when faced with the life-threatening situations.

Briefly, profiles that exhibited extensive biting were grouped under fight and labeled as types of aggression. Those in which wing flapping rate was high were considered to be flight responses. The remaining profiles exhibiting extensive use of distress calls without any or minimal biting were grouped under “seek” and labeled as exhibiting various levels of alarm. One group expressed helplessness by “freezing” without any emission of distress calls or fighting behavior. Another exhibiting mixed expression of multiple behaviors, suggested “confusion” about how to act during distress. Both of these clusters were grouped under “fright”. Each behavior represented a viable personality trait of an individual. The ten categories of response profiles were labeled and defined as follows:

1. **Fearful helplessness**: This fright-behavior consisted of freezing, and was expressed by the juveniles. Freezing was expressed during the arrest phase of predator-induced distress when the animal did not have a possibility of escaping.
2. **Confusion**: A lack of a clear and specific escape strategy by the animal was indicative of "confusion" as the animal tried everything to escape. These fright-behaviors included a combination of emission of echolocation and/or distress sounds as well as wing flapping and biting activity. Different animals exhibited a subset of these escape responses under the three conditions tested.
3. **Aggression-defiance**: Defiant aggression represents a response profile where the animals mostly focused on biting with very little wing flapping and a relatively low level of emission of echolocation pulses.
4. **Aggression-escape**: Active aggressive activity while trying to escape represented a fight mechanism with a relatively low rate of emission of echolocation pulses and a medium to high tendency to indulge in biting that was on average higher than the frequency of wing flapping.
5. **Aggression-vigilance**: Aggression with vigilance represented a fight mechanism in which bats emitted echolocation pulses at a high rate. As a group, these individuals exhibited some wing flapping and a relatively high rate of biting. Animals exhibiting any category of aggressive fight tactic did not emit any distress calls.
6. **Escape-quiet**: Quiet escape represented a “flight” or fleeing type of response profile that consisted of wing flapping together with a very low rate of emission of echolocation pulses. This profile also included a small increase in the frequency of biting.
7. **Escape-aggressive**: Aggressive escape represented another fleeing tactic that consisted of more than twice the level of emission of echolocation pulses (compared to quiet escape) and a trajectory of increased wing flapping accompanied by biting behavior.
8. **Alarm-low**: Escape behaviors labeled as "seek" were those in which distress calls were emitted either as single syllables or multiple syllables within a train. Low alarm was characterized by emission of a high number of syllables within a call (train of syllables) even though their rate of emission was low. As a group, bats in this category exhibited only occasional or no biting.
9. **Alarm-moderate**: A moderate alarm profile within the “seek” group was characterized by emission of both a high distress call rate and a high number of syllables within a call. Biting was minimal.
10. **Alarm-high**: This alarm response profile for seeking help was characterized by the highest distress call rate as well as a relatively high number of syllables present within each call. Wing flapping and biting were absent.

Classification of the behavioral response profiles within a *k*-means cluster plot (see parallel coordinate plots in Fig 4) revealed that each of the two divergent trajectories of cluster locations in a two-dimensional plot was associated with a different overall coping strategy. One direction reflected social dependence for escape from life threatening distress and consisted largely of the production of increasingly intense distress calls. The other direction was associated with self-reliant escape mechanisms consisting of wing flapping, echolocating and biting.

### Context-sensitivity of escape strategies

**Fig 5:**
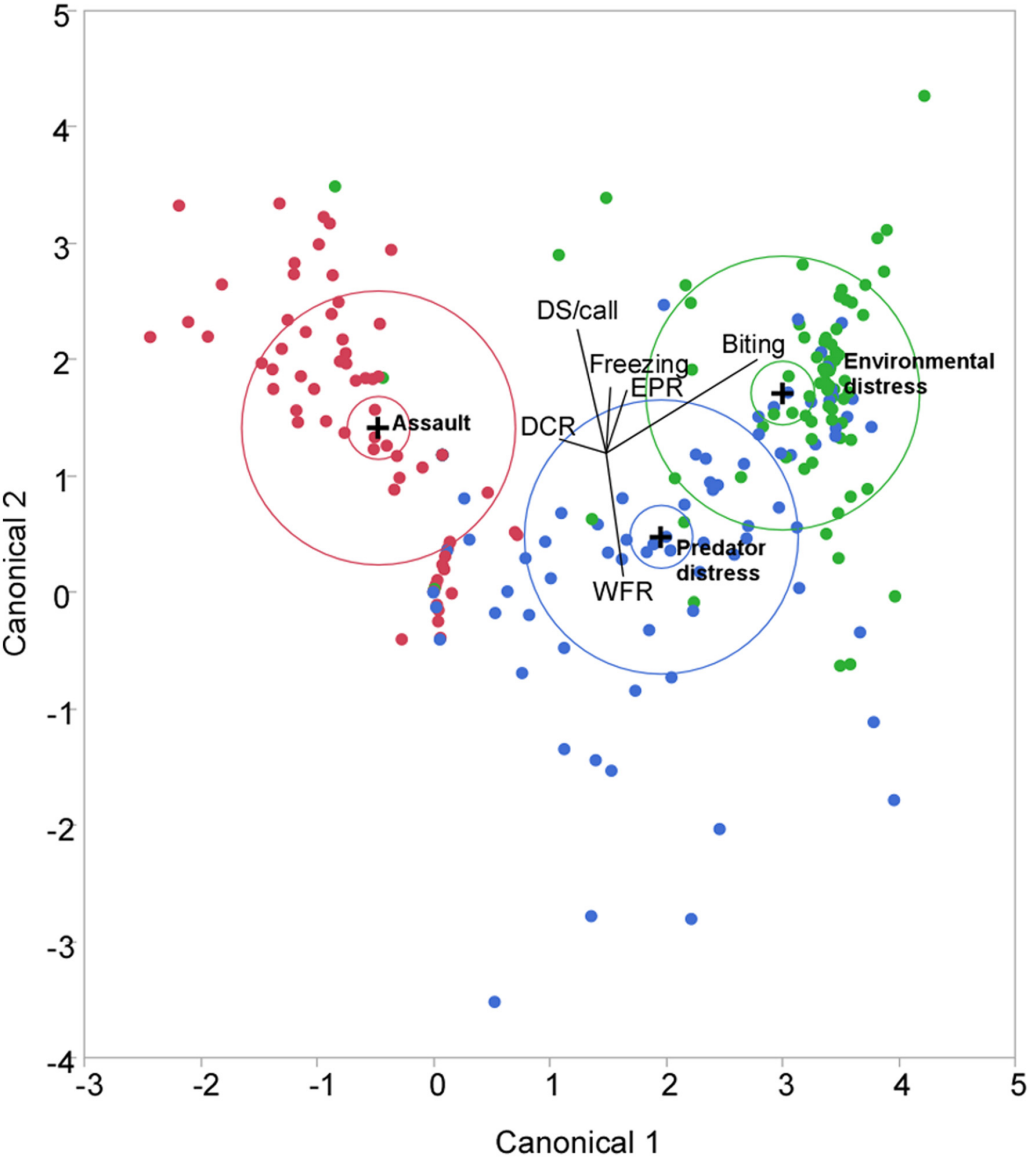
Scatterplot of behavioral response profiles of juveniles and adults within two-dimensional canonical space for environmental distress (green dots), predator distress (blue dots) and arrest (red dots). Variable direction rays are placed within the plot. The "+" symbols indicate the location of the multivariate mean. The canonical plot shows the points and multivariate means in the two dimensions that best separate the groups. The size of the inner ellipse corresponds to a 95% CL for the mean. Groups that are significantly different tend to have non-intersecting ellipses, in this case suggesting significant differences among the three life-threatening distressful situations encountered by the bats. The outer ellipses show areas that contain roughly 50% of the points for that group.

When trapped in a mist net (environmental threat) and during predator approach (predator threat) big-footed myotis showed more frequent biting and wing flapping behavior than during arrest (Tables 1 and 2; Fig 5). However, biting propensity was highest during environmental threat, and wing flapping rate was highest during predator threat; these results weren’t significant for juvenile bats (Tables 1 and 2; Fig 5). Moreover, bats emitted more echolocation pulses both during environmental and predator threat compared to arrest, especially during environmental threat (Tables 1 and 2; Fig 5).

**Table 2:** Comparisons of escape behavior of big-footed myotis among different contexts.

Behavioral variables	Age	Test	Test value	*P*	Post hoc	Env-Pre	Env-Arr	Pre—Arr
Proportion of time biting (%)	Adult	Friedman	98.16 (*m* = 3, *N* = 54)	**< 0.001**	Dunn's	**< 0.001**	**< 0.001**	**< 0.001**
	Juvenile	Friedman	34.53 (*m* = 3, *N* = 28)	**< 0.001**	Dunn's	NS	**< 0.001**	**< 0.05**
Number of flapping/second	Adult	Friedman	52.79 (*m* = 3, *N* = 54)	**< 0.001**	Dunn's	**<0.05**	**< 0.001**	**< 0.001**
	Juvenile	Friedman	15.04 (*m* = 3, *N* = 28)	**0.001**	Dunn's	NS	NS	**< 0.01**
Number of pulses/ second	Adult	Friedman	41.97 (*m* = 3, *N* = 54)	**< 0.001**	Dunn's	**< 0.05**	**< 0.001**	**< 0.001**
	Juvenile	Friedman	14.82 (*m* = 3, *N* = 28)	**0.001**	Dunn's	NS	**< 0.01**	NS
Number of calls/second	Adult	Friedman	50.80 (*m* = 3, *N* = 54)	**< 0.001**	Dunn's	NS	**< 0.001**	**< 0.001**
	Juvenile	Friedman	32.93 (*m* = 3, *N* = 28)	**< 0.001**	Dunn's	NS	**< 0.01**	**< 0.001**
Syllable frequency/call	Adult	Friedman	48.81 (*m* = 3, *N* = 54)	**< 0.001**	Dunn's	NS	**< 0.01**	**< 0.001**
	Juvenile	Friedman	29.73 (*m* = 3, *N* = 28)	**< 0.001**	Dunn's	NS	**< 0.01**	**< 0.001**
% exhibiting freezing behavior	Adult	Pearson chi-square	23.86 (*df* = 2)	**< 0.001**	Fisher exact	NS	**< 0.001**	**< 0.01**
	Juvenile	Pearson chi-square	1.95 (*df* = 2)	0.378	-	-	-	**-**

Statistically significant results are highlighted in bold. Env refers to environmental threat, Pre refers to predator threat, Arr refers to arrest. ‘-’ indicates that no post hoc comparison was made.

Linear discriminant analysis (LDA) revealed a significant difference (Wilks’ Lambda < 0.001; no misclassification) in how animals responded to the particular type of distressful situation they encountered (Fig 5). This analysis provides a means to test goodness-of-fit of the regression model adopted to appropriately classify response parameters into the categories identified by *a priori* clustering method. If multiparametric values match a category that is different from the one assigned by the clustering algorithm, then this analysis counts it as a misclassification. A high degree of misclassification can cast doubt on the appropriateness of clustering within multidimensional space. There was some overlap of 50% contours for environmental and predator induced distress. The reaction to arrest was more distinct, particularly because it rarely resulted in a biting response. A similar separation was obtained for both juveniles and adults. A fifth (21.14%) of the response profiles were misclassified and the arrest condition showed the best fit per the maximum area under the curve for the receiver-operator characteristic or ROC plot. An ROC curve is a graphical plot that illustrates the performance of a binary classifier system as its discrimination threshold setting is varied. The area under the ROC plot identifies the probability of hitting or missing the target classification. ROC analysis is also related in a direct and natural way to cost/benefit analysis of diagnostic decision-making given a rational choice. For our analysis, ROC analysis provided an additional tool to confirm the applicability of context as an optimal model for minimizing cost by making a “correct” decision.

For the overall data, arrest frequently resulted in the production of distress calls whereas predator approach never resulted in the production of distress calls, but frequently resulted in biting at the net. As expected, biting was most frequent (mean biting duration of 52%) during environmental distress when bats were entangled in the net. During arrest, more echolocation pulses were recorded by microphone 4 when the trapped bats emitted distress calls. In contrast, when the affected bats either emitted echolocation pulses or remained motionless, the number of echolocation pulses recorded at the same microphone were far fewer (One way ANOVA, *F*_2,61_ = 5.70, *P* = 0.011).

### Age and sex differences

#### Multivariate analysis of response profiles

**Fig 6:**
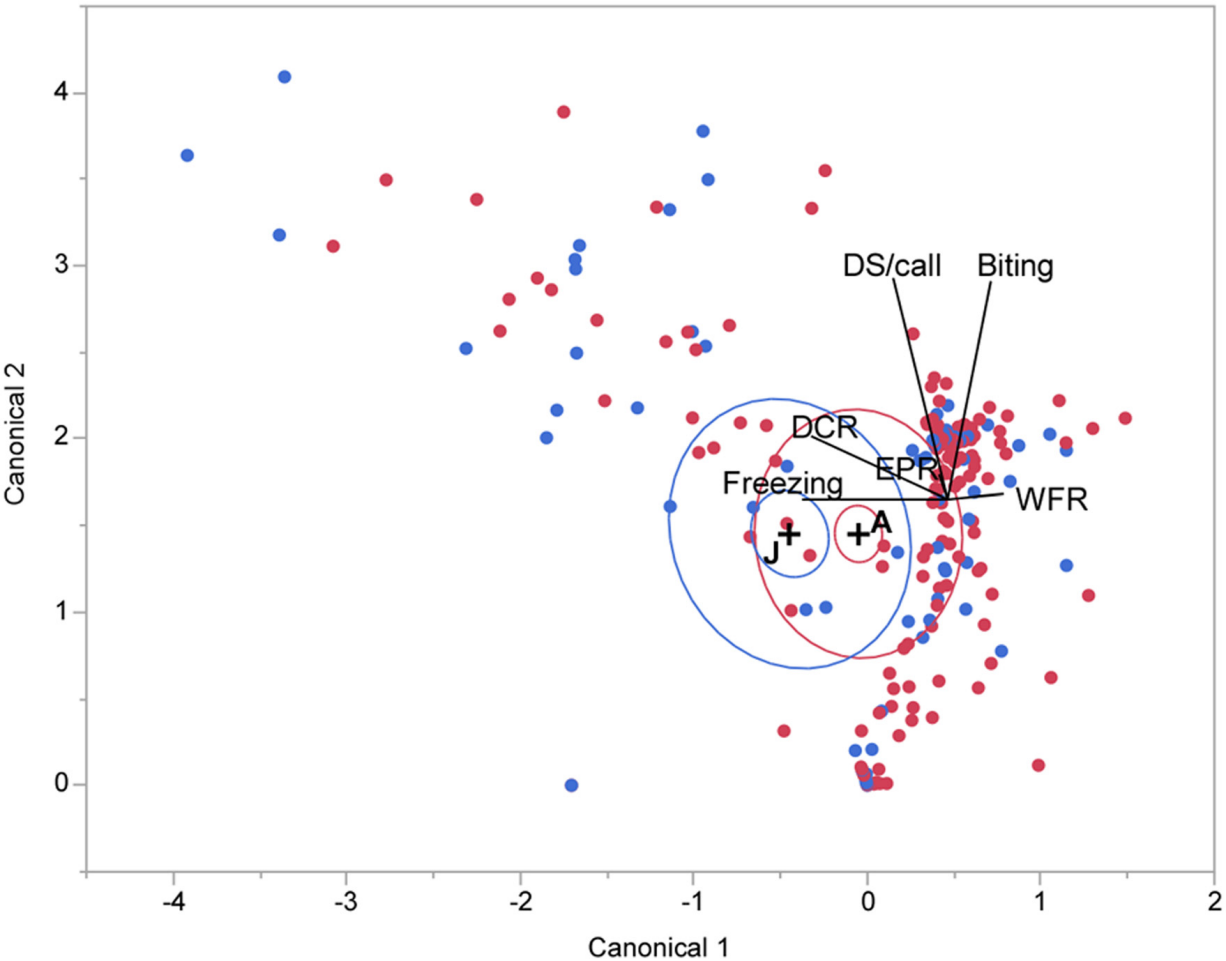
Scatterplot of behavioral response profiles of juveniles (blue dots) and adults (red dots) within two-dimensional canonical space. Groups that are significantly different tend to have non-intersecting ellipses, in this case suggesting significant differences between juveniles and adults. See Fig 5 for details on the meaning of plot symbols.

Both adults and juveniles exhibited the same set of response profiles illustrated by parallel coordinate plots in Fig 5 and as explained above. The allocation ratios (for percent expressed within each group), however, were different for adults vs. juveniles (see Figs 5 and 6). Overall, a relatively greater percentage of juveniles expressed fearful and assistance-seeking profiles whereas a greater percentage of adults expressed exhibited self-reliant (either fight or flight) strategies.

We used LDA to test whether the mean response profiles of groups distinguished by age or sex were significantly different. Hierarchical clustering revealed a greater misclassification of juvenile behavioral profiles than those of adults. Escape response profiles of adults were diverse but distinct, showing virtually no misclassification with response profiles exhibited by juveniles (Fig 6). Sex differences, tested only in juveniles, while present were not significant.

A canonical plot of the first two most correlated, weighted combinations of orthogonally projected variables revealed nonoverlapping 95% CL ellipses around the centroids. A model fit of wing-flapping, distress calls (DCR) and freezing produced a significant effect (Wilks’ Lambda = 0.049; no misclassification) of age on the escape response profile, but this effect became less than significant when all six parameters were included (Wilks’ Lambda = 0.220). The echolocation pulse rate changed the least with age, which is not surprising given that both juveniles and adults need to scan their environment to determine an escape strategy. A linear classification method resulted in a misclassification of 34% of the data. A canonical plot of the data in two dimensions showed the 95% CL ellipses around the multivariate means for juvenile and adults to be separate, although the 50% contours (containing roughly 50% of the points) showed substantial overlap. Consistent with the cluster analysis, these data suggest that both juveniles and adults expressed similar behaviors. As indicated by the ray plot within the canonical space, however, there was separation between the two groups along the freezing-wing flapping axis.

#### Multiple comparisons

*Post hoc* multiple comparisons of different parameters showed that adult big-footed myotis showed more violent biting during environmental threat, and a significantly higher wing-flapping rate during both environmental and predator threat compared to juveniles (Tables 1 and 3; Fig 6). Furthermore, juvenile bats included a higher percentage of individuals exhibiting freezing compared to adults, except during arrest (Tables 1 and 3). During arrest, more adult bats remained motionless than during environmental or predator threat (Tables 1 and 2).

**Table 3:** Comparisons of escape behavior of big-footed myotis between adults and juveniles during three contexts.

Behavioral variables	Environmental threat		Predator threat		Arrest	
	Test value	*P*	Test value	*P*	Test value	*P*
Proportion of time biting (%)	*U* = 539.5 (*N*_*1*_ = 54, *N*_*2*_ = 28)	**0.022**	*T* = -1.553 (*df* = 80)	0.124	*U* = 743.0 (*N*_*1*_ = 54, *N*_*2*_ = 28)	0.793
Number of flapping/second	*U* = 528.5 (*N*_*1*_ = 54, *N*_*2*_ = 28)	**0.025**	*U* = 553.0 (*N*_*1*_ = 54, *N*_*2*_ = 28)	**0.047**	*U* = 741.0 (*N*_*1*_ = 54, *N*_*2*_ = 28)	0.871
Number of pulses/second	*U* = 700.0 (*N*_*1*_ = 54, *N*_*2*_ = 28)	0.583	*U* = 663.5 (*N*_*1*_ = 54, *N*_*2*_ = 28)	0.359	*U* = 702.5 (*N*_*1*_ = 54, *N*_*2*_ = 28)	0.502
Number of calls/second	*U* = 704.0 (*N*_*1*_ = 54, *N*_*2*_ = 28)	0.220	-	-	*U* = 563.0 (*N*_*1*_ = 54, *N*_*2*_ = 28)	**0.048**
Syllable frequency/call	*U* = 701.0 (*N*_*1*_ = 54, *N*_*2*_ = 28)	0.195	-	-	*U* = 606.5 (*N*_*1*_ = 54, *N*_*2*_ = 28)	0.123
% exhibiting freezing behavior ^a^	—	**0.014**	—	**0.006**	—	0.582

N_1_ refers to the number of adult bats.

N_2_ refers to the number of juvenile bats.

Statistically significant results are highlighted in bold.

‘-’ indicates that big-footed myotis didn’t emit distress calls during predator threat. The differences in escape response between adults and juveniles were compared using Independent-Sample T test or Mann-Whitney U test.

^a^ These statistical analyses were performed with Fisher's exact test, thus, there was not test value given.

With respect to vocalizations, we did not detect significant differences in echolocation pulse rate between adults and juveniles within any context (Tables 1 and 3). Distress calls were absent during predator approach, but emitted at the highest rate during arrest. Among these, juvenile bats emitted more distress calls than adults (Tables 1–3). No significant age-related difference, however, was observed in the number of syllables emitted per call, irrespective of the type of threat (Tables 1 and 3). Also, the number of distress calls emitted did not differ significantly between juvenile and adults exposed to either environmental or predator threat (Tables 1 and 3).

## Discussion

To maximize expected fitness, prey species have evolved a wide range of escape tactics, such as fleeing, fighting, remaining immobile, and emitting antipredator calls [43,44,45,46]. Bats’ fitness relies greatly on their unabated ability to forage over geographically widespread habitats. This is largely a result of their nocturnal habits, group living, flight and echolocation ability, which either deter or minimize contact with potential predators [47]. These evolutionary adaptations partly account for bats’ global distribution [48,49]. Additional behavioral adaptations to avoid predation appear to be unnecessary. However, there is potential for predation of bats by a number of mammalian, including human, avian and even amphibian species [13]. Therefore, the presumed absence of antipredator responses in bats was questioned recently, but evidence in support of their presence remained equivocal [13].

Quantifying behavioral manipulations and observations in the field always has a certain element of unreliability. Multivariate classifiers, however, have the power to overcome variation from random sources of error in the dataset to reveal potentially interesting patterns in the data. In this study, we obtained two important results. Our results are based on the assumption that capturing animals for ecological studies transiently mimics predation risk [50,51]. First, we discovered that during life threatening distress, big-footed myotis behave in a context-dependent manner despite their evolutionary adaptations for predator avoidance. This contradicted our null hypothesis. Second, our results supported our hypothesis (at the 0.05 significance level) regarding the presence of the same overall escape response profiles in both adults and juveniles. We did observe a significant effect of age, however, for a subset of behaviors adopted by adults vs. juveniles and a trend for differences in the overall response profile data as well. In brief, adults were decisively more aggressive (exhibiting biting along with wing flapping behavior more frequently) than juveniles. These differences were consistent with the divergent trajectories indicated by multivariate response profile clusters (independent of context and age) that nevertheless separated juvenile-like from adult-like response profiles (see Fig 3).

### Escape behaviors

#### Freezing and confusion

Choosing an appropriate behavioral strategy for escape is important for increasing the likelihood of survival without incurring excessive costs [39,52,53]. Remaining motionless can reduce the probability of being detected by a predator, and thus increase prey survival [54]. For instance, the harvestman, *Eumesosoma roeweri*, reduce the chances of being recognized as prey items by remaining completely motionless in the presence of a predator [55]. Feigning death by becoming motionless has been considered as a common defense mechanism in a number of taxa, including mammals, birds, fishes, amphibians, reptiles and insects [45]. Death-feigning is a state of hypnotic reaction or immobility elicited by external stimuli [56], usually interpreted as a last-resort antipredator tactic [57]. In particular, death-feigning may decrease predator’s interest to prey, or make predator believe that the prey is successfully controlled decreasing its motivation attack [58]. Only a few individuals of big footed myotis exhibited freezing and this behavior was observed mostly during arrest (see Tables 1 and 2). We consider these individuals as expressing fearful helplessness.

A separate group of individuals exhibited a mixed response profile consisting of an unusual combination of escape-related responses expressed across all contexts. For example, individuals in this group indulged in moderate levels of echolocation, communication, as well as wing flapping and biting behaviors. We consider these bats to be overwhelmed by fear and try everything to escape resulting in a fusion of multiple actons. This is consistent with given the location of this profile at the base of the two divergent behavioral trajectories identified in the cluster plot in Fig 3. This response profile was expressed by an equal proportion of individuals from the unequal samples of adults and juveniles (see Fig 4). It is possible that, regardless of age, a response profile characteristic of fight or flight has not yet crystalized in these individuals.

#### Relying on others: alarm

Animals usually emit antipredator calls (alarm calls and mobbing calls), and adjust the structures and parameters of calls according to the threat level and the situation within which it is encountered with predators [22,59]. Distress calls are an important component of antipredator calls [43]. Big footed myotis, however, never or rarely emitted distress calls prior to arrest. Similar findings have been reported in tufted titmice, *Baeolophus bicolor* [51]. It is possible that prey face higher predation risk due to the extreme cost of antipredator calls for eavesdropping by predators. Some reports point out that alarm calls not only warn conspecifics of the danger, but also attract the predator’s attention [60]. Mobbing calls of pied flycatcher, *Ficedula hypoleuca*, attract the predator to move to their nest, and increase the risk of nest predation [61]. During arrest, eavesdropping of predators on distress calls may result in excessive cost to big footed myotis. Thus, distress calls might prematurely expose bats trapped in the mist net and during perceived threat. Hence, it is not surprising that big footed myotis emitted more distress calls during arrest compared with pre-encounter contexts.

#### Relying on self: fight or flight

During physical distress, animals are in considerable danger and may allocate all of their time and energy to either defense or escape-related efforts, such as biting, flapping, kicking, death-feigning and emitting distress calls [19,52,62,63,64]. Most animals fight back (e.g. biting, kicking, wriggling) and attempt to escape (e.g. wing-flapping) immediately after being captured [65,66]. Perrone [65] proposed that active fighting and attempts to escape may increase the survival chance for prey that are nearer to their predators in size, strength or weaponry. Alternatively, animals may choose to fight back or engage in attention avoidance strategies by becoming motionless (“freeze”), especially when confronted with a situation of imminent danger from which there is no escape [67], such as the one encountered during predator approach in the context of our study.

Unlike distress calls, echolocation pulses attenuate rapidly, are highly directional and require a large amount of metabolic energy when emitted in a non-foraging context [30,68]. Big footed myotis emitted mainly echolocation pulses during nonspecific environmental distress (entanglement in the mist-net and predator approach). Even though the mostly ultrasonic echolocation vocalizations are unlikely to be heard by predators, they do have some communicative potential and may be used to transfer risk signals to conspecifics [69]. It is more likely, however, that similar to dolphins, their emission is associated with increased vigilance, allowing them to rapidly and constantly scan their immediate environment [70,71,72]. Further experiments are necessary to establish the exact role of echolocation pulses emitted during distress.

Echolocation pulses, however, are not a good communication signal relative to distress calls, which are optimized for long-distance communication [73], and may serve multiple functions, including requesting aid from kin or unrelated altruists, warning conspecifics of the presence or location of a predator, startling the predator into releasing the caller, and attracting secondary predators which distract the attacking predator [52]. This was borne out by our analysis of recordings during arrest of echolocation pulses at the microphone (microphone 4) pointing away from the net, which registered siginificantly more echolocation pulses, presumably from neighboring bats, in conjunction with distress calls compared to other conditions. This likely means that more bats were attracted by the emission of distress calls [30].

### Age-related differences in defense/escape strategies

An individual’s ability to escape from a predator may depend greatly on its bite force, locomotor ability and experience [28,46]. Given their smaller body size, lower performance ability and lack of experience, juvenile tegu lizards, *Tupinambis merianae*, tend to exhibit escape responses or rely on assistance from others when faced with life-threatening distress, whereas adults often adopt aggressive defensive postures when they encounter a predator [46]. Another example is that of leopard gecko, *Eublepharis macularius*, which exhibits clear ontogenetic changes of antipredator tactics, from a threat–vocalization–bite strategy prevailing in juveniles to an escape strategy typical for adults [74]. In the playback experiment of alarm calls, juvenile Willow Tits, *Poecile montanus*, more often stayed motionless or moved shorter distances than adults [28]. In our study, more juveniles than adults remained motionless within environmental and predator threat contexts.

For our multivariate analysis, freezing, wing flapping and distress call rate (but not number of syllables/call) were the most affected by age. Juvenile big footed myotis have smaller body size, lower body mass and weaker flying ability than adults [36,75], and they may have never encountered predators prior to this study. Therefore, vulnerable juveniles are potentially at a disadvantage compared with adults. Thus, it is not surprising that juveniles did not exhibit biting and wing flapping behavior as frequently as adults during environmental and predator threat, but emitted more distress calls during arrest. During arrest (capture by a predator), distress calls significantly increase the probability of escape from predators [76,77], which may explain the use of distress calls by juveniles of big footed myotis.

### Coping with distress

Our results show that bats exhibit a statistically different coping profile when faced with life threatening distress under different contexts. Relatively low levels of fear, as exemplified by nonspecific threats from the environment (entanglement in the mist-net in our study), promote pre-encounter defenses, such as meal-pattern reorganization and protective nest maintenance [78]. Given the diversity of behaviors, it is likely that multiple brain regions are involved, some of which are well studied within the context of decision making [31]. Fear and its context are clearly relevant, suggesting the involvement of the limbic brain, amygdala and the hippocampus in particular, whose role within a fear context is now fairly well established. For example, the central amygdaloid nucleus, dorsal hippocampus, entorhinal cortex and ventral periaqueductal gray were labeled by c-fos immunoreactivity (cellular marker for neural activity) in the rat brain following freezing responses [79,80].

The fact that bats can rapidly switch between different behavioral strategies with situational changes during life-threatening distress is also interesting from a neurological perspective. This may be explained by the ability of the frontal cortex (a higher control center), to selectively activate different constellations of neural circuits involved in expressing different escape and other antipredator strategies. Since the frontal cortex continues to develop after birth [81], it is also the optimal, but not necessarily the only region for encoding socially and environmentally determined changes in neural wiring and/or synaptic efficacy that may underlie age-related differences observed in our study.

In summary, we provide a first, experimentally determined and statistically tested, multiparametric classification of escape strategies in bats. Our results together with studies of others show that bats have a keen sense to make ergonomically and ecologically relevant decisions when subjected to life threatening distress. These decisions become modified somewhat with age and learning, which is substantiated by neurological studies. Studies in other species, including humans, have identified specific brain regions involved in making these decisions and big-footed myotis may be an excellent animal model to obtain a deeper understanding of the neural basis of their ability to choose between and modify their escape strategies. This is one of the first studies to show how echolocation and communication abilities of bats are integrated adaptively, while still supporting individual variability, within escape behaviors.
